# Trends in Heart Disease Mortality among Mississippi Adults over Three Decades, 1980-2013

**DOI:** 10.1371/journal.pone.0161194

**Published:** 2016-08-12

**Authors:** Vincent L. Mendy, Rodolfo Vargas, Lamees El-sadek

**Affiliations:** Office of Health Data and Research, Mississippi State Department of Health, Jackson, Mississippi, United States of America; Garvan Institute of Medical Research, AUSTRALIA

## Abstract

Heart disease (HD) remains the leading cause of death among Mississippians; however, despite the importance of the condition, trends in HD mortality in Mississippi have not been adequately explored. This study examined trends in HD mortality among adults in Mississippi from 1980 through 2013 and further examined these trends by race and sex. We used data from Mississippi Vital Statistics (1980–2013) to calculate age-adjusted HD mortality rates for Mississippians age 25 or older. Cases were identified using underlying cause of death codes from the International Classification of Diseases, Ninth Revision (ICD-9: 390–398, 402, 404–429) and Tenth Revision (ICD-10), including I00-I09, I11, I13, and I20-I51. Joinpoint software was used to calculate the average annual percent change in HD mortality rates for the overall population and by race and sex. Overall, the age-adjusted HD mortality rate among Mississippi adults decreased by 36.5% between 1980 and 2013, with an average annual percent change of -1.60% (95% CI -2.00 to -1.30). This trend varied across subgroups: HD mortality rates experienced an average annual change of -1.34% (95% CI -1.98 to -0.69) for black adults; -1.60% (95% CI -1.74 to -1.46) for white adults; -1.30% (95% CI -1.50 to -1.10) for all women, and -1.90% (95% -2.20 to -1.50) for all men. From 1980 to 2013, there was a continuous decrease in HD mortality among adult Mississippians. However, the magnitude of this reduction differed by race and sex.

## Introduction

In recent decades, heart disease (HD) mortality has decreased in the United States (U.S.); however, HD remains the leading cause of death among U.S. adults [1]. HD mortality rates in Mississippi (240.0 deaths per 100, 000 population) are higher than the national average (169.8 deaths per 100,000 population) in 2013 [2]. In 2013, HD accounted for 25% of all deaths among Mississippi adults, and blacks and males were disproportionally affected [3]. Researchers have observed similar national-level declines in coronary heart disease (CHD) from 1980–2000 [4] and in the Atherosclerosis Risk in Communities (ARIC) Study which includes the city of Jackson, Mississippi from 1987–2008 [5]. In addition, decreases in CHD have been observed in other countries and regions, such as Australia [6], England and Wales [7], Sweden [8], Turkey [9], and the European Union [10]. Researchers have attributed these declines to reductions in major risk factors for HD (e.g., cigarette smoking, hypertension, and hyperlipidemia) and evidence-based medical and pharmaceutical therapies that improve disease control (e.g., individuals with hypertension managing their blood pressure) [11,12]. Knowledge of trends in HD mortality can encourage the development of effective prevention and intervention strategies, support public health policies aimed at reducing disparities in HD mortality, and facilitate the achievement of the Healthy People HD objective [13]. However, no previous studies have described annual changes in HD mortality rates in Mississippi. To address this gap, the current study calculated the average annual percent change (AAPC) in age-adjusted HD mortality rates among Mississippi adults (≥ 25 years) between 1980 and 2013. In addition, we examined how AAPC varied by race and sex.

## Materials and Methods

The numbers of adult (≥25 years of age) deaths due to HD for each year from 1980 through 2013 were extracted from Mississippi Vital Statistics (available years of data) [3]. We used underlying cause of death codes from the International Classification of Diseases, Ninth Revision (ICD-9: 390–398, 402, 404–429) and Tenth Revision (ICD-10), including I00-I09, I11, I13, and I20-I51, to identify cases [14–16]; ICD-9 was used for the years 1980 through 1999 and ICD-10 was used for later years. We then used Mississippi population census counts to calculate age-adjusted HD mortality rates and standard errors for the overall population, by race (blacks, whites), by sex (women, men), and by race and sex (black women, black men, white women, white men) in SAS 9.4 (SAS Institute Inc.). Age-adjustment was performed using the direct method and the 2000 U.S. standard projected population [8]. Next, we exported age-adjusted HD mortality rates and standard errors to the U.S. Surveillance, Epidemiology, and End Results (SEER) Joinpoint software (4.1.1.5) (http://surveillance.cancer.gov/joinpoint/) to calculate the average annual percent change (AAPC) in HD mortality rates for the overall population, by race, by sex, and by race and sex. Joinpoint regression analysis identifies trend breaks (joinpoints) or points of significant change in a trend [10, 15]. This analysis identified time periods with statistically distinct log-linear trends in HD mortality rates [10]. Using a Bayesian information criterion approach to select the most parsimonious model of best fit, we specified a maximum of three joinpoints [7, 8, 10, 17]. The slopes of the models were used to calculate the annual percent change (APC) for each trend segment and the AAPC (the weighted average of the APC) [15]. For each AAPC, 95% confidence intervals (CIs) were calculated and tested to determine whether there was a significant difference from the null hypothesis of no change (0%) [18, 19] using a p-value of <0.05. This investigation was approved by the Mississippi State Department of Health Institutional Review Board.

## Results and Discussion

During the 33-year period from 1980 through 2013, the age-adjusted HD mortality rate for Mississippi adults decreased by 36.5%, and the AAPC was -1.60% (95% CI, -2.00 to -1.30).

**Fig 1:**
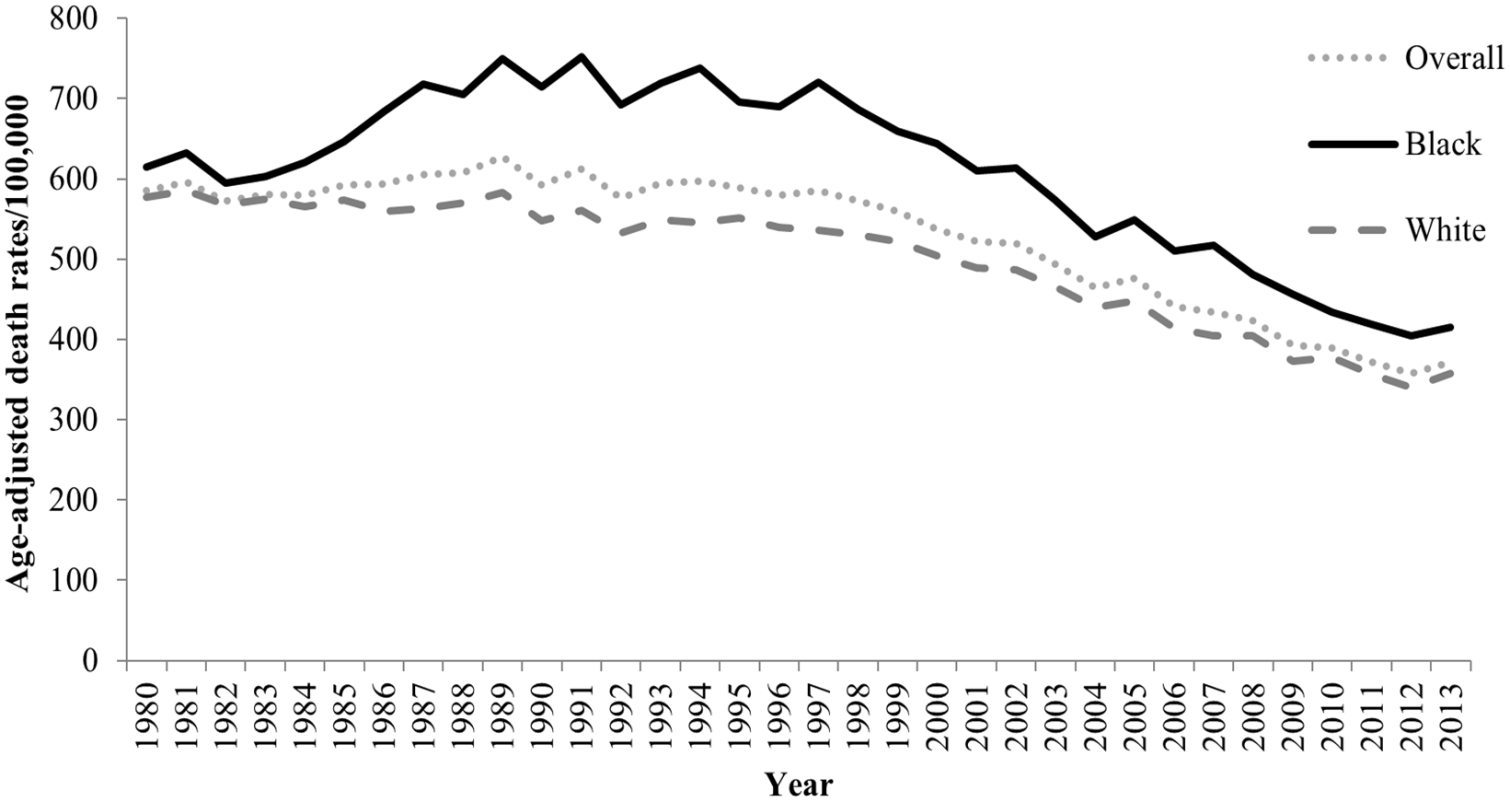
Trends in age-adjusted HD mortality rates for Mississippi adults ≥25 years by race, 1980 through 2013.

**Fig 2:**
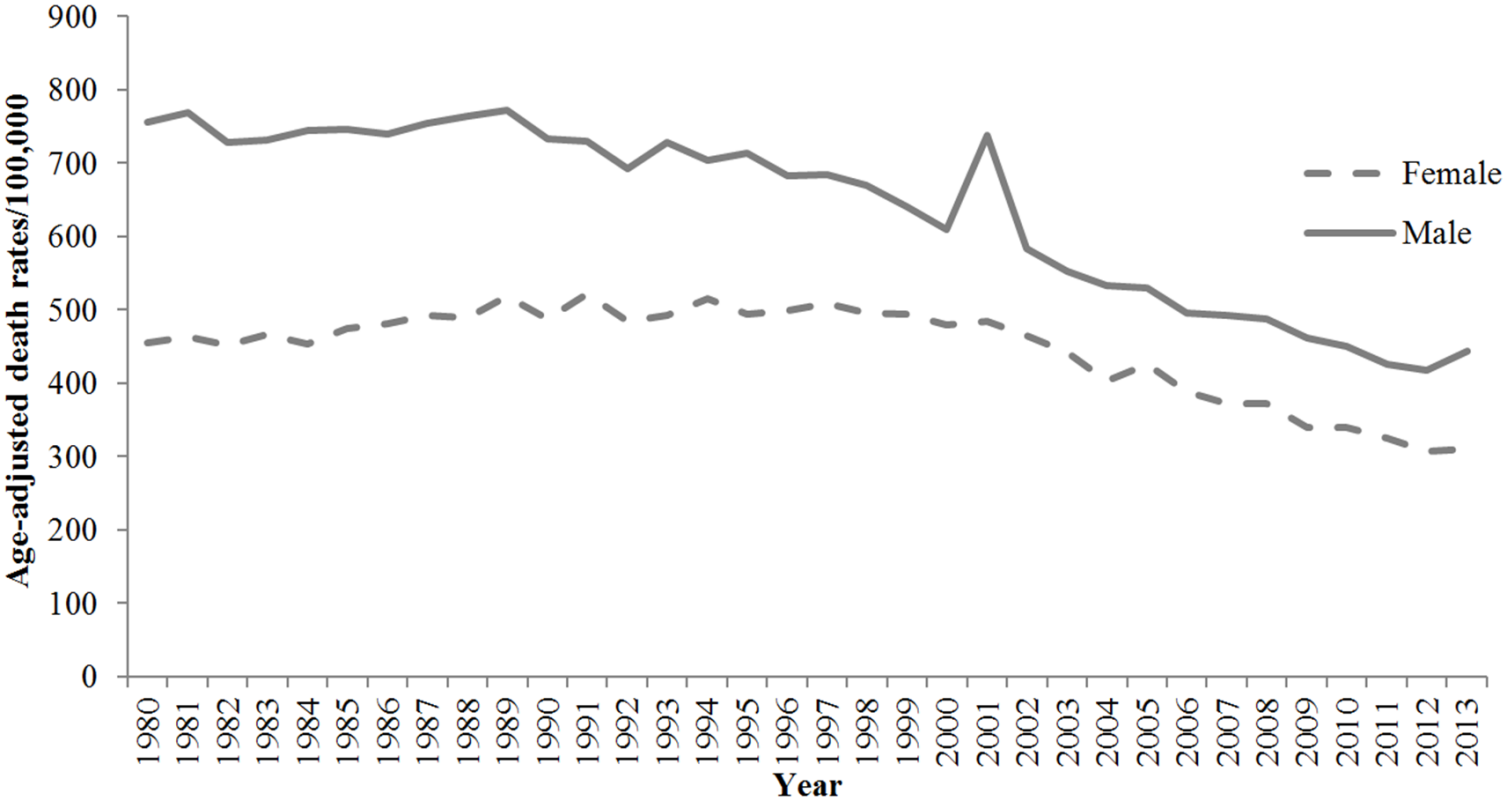
Trends in age-adjusted HD mortality rates for Mississippi adults ≥ 25 years by sex, 1980 through 2013.

**Fig 3:**
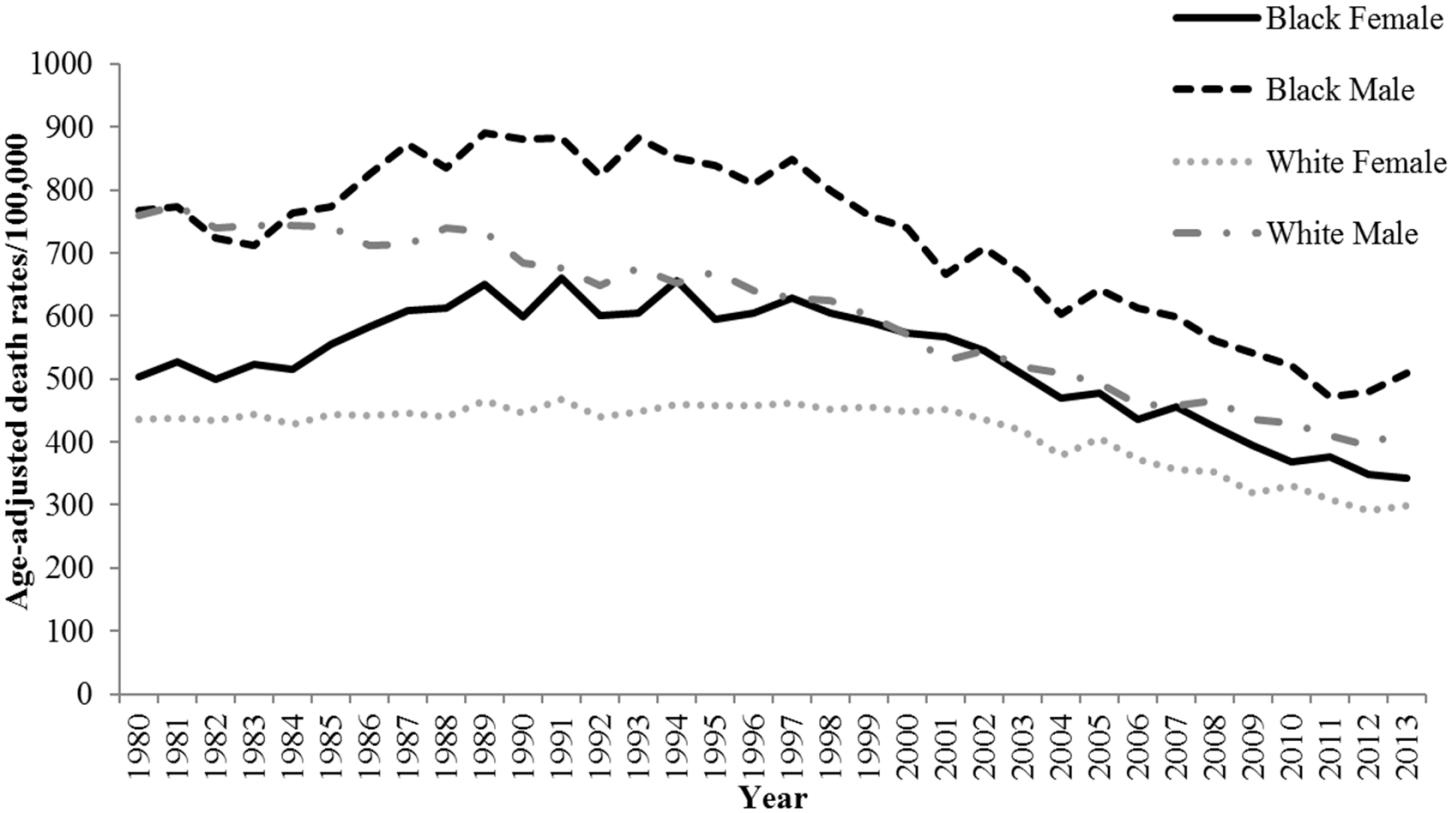
Trends in age-adjusted HD mortality rates for Mississippi adults ≥25 years by race and sex, 1980 through 2013.

These trends varied by both race and gender. Among black adults, the HD mortality rate decreased by 32.5%, and the AAPC was -1.34% (95% CI, -1.98 to -0.69). Among white adults, the HD mortality rate decreased by 38.1%, and the AAPC was -1.60% (95% CI, -1.74 to -1.46). Among women, the HD mortality rate decreased by 31.6%, and the AAPC was -1.30% (95% CI, -1.50 to -1.10). Among men, the HD mortality rate decreased by 41.4%, and the AAPC was -1.90% (95% CI, -2.20 to -1.50). The results for race-gender subgroups reveal further patterns. Among black women in Mississippi, the HD mortality rate decreased by 31.7% between 1980 and 2013, and the AAPC was -1.10% (95% CI, -1.50 to -0.70). Among white women, the HD mortality rate decreased by 31.5%, and the AAPC was -1.20% (95% CI, -1.40 to -1.10). Among black men, the HD mortality rate decreased by 33.5%, and the AAPC was -1.50% (95% CI, -2.02 to -0.80). Finally, among white men, the HD mortality rate decreased by 44.4%, and the AAPC was -2.00% (95% CI, -2.20 to -1.80) (Table 1, Figs 1–3, S1–S9 Figs).

**Table 1:** Trends in heart disease age-adjusted death rates among Mississippi adults 25 years and older, 1980–2013: Joinpoint analysis^a^. CI, confidence interval; APC, annual percent change; AAPC, average annual percent change.

	Trend 1			Trend 2			Trend 3			Trend 4			1980–2013	
	Years	APC^b^	95% CI	Years	APC	95% CI	Years	APC	95% CI	Years	APC	95% CI	AAPC^c^	95% CI
Overall	1980–1997	-0.35	-0.60,-1.00	1997–2009	-3.47	-4.0,-3.0	2009–2013	-1.51	-3.9,1.0	--	--	--	-1.60^d^	-2.0,-1.3
Black	1980–1983	-1.62	-5.75,2.69	1983–1987	5.03	0.78,9.46	1987–1997	-0.16	-0.87,0.56	1997–2013	-3.54	-3.86,-3.23	-1.34^d^	-1.98, -0.69
White	1980–1998	-0.44	-0.63,-0.26	1998–2013	-2.97	-3.22,-2.73	--	--	--	--	--	--	-1.60^d^	-1.74, -1.46
Females	1980–1999	0.52	0.27,0.76	1999–2013	-3.69	-4.07,-3.31	--	--	--	--	--	--	-1.30^d^	-1.50, -1.10
Males	1980–1997	-0.50	-0.98,-0.03	1997–2013	-3.30	-3.83,-2.77	--	--	--	--	--	--	-1.90^d^	-2.20, -1.50
Black Females	1980–1989	2.89	1.93,3.86	1989–1999	-0.45	-1.31,0.42	1999–2013	-4.04	-4.51,-3.56	--	--	--	-1.10^d^	-1.50, -0.70
White Females	1980–2000	0.28	0.09,0.47	2000–2013	-3.52	-3.87,-3.16	--	--	--	--	--	--	-1.20^d^	-1.40, -1.10
Black Males	1980–1983	-1.95	-6.95,3.31	1983–1989	3.50	1.17,5.89	1989–1997	-1.12	-2.42,0.21	1997–2013	-3.36	-3.76,-2.95	-1.50^d^	-2.02,-0.80
White Males	1980–1995	-1.05	-1.37,-0.73	1995–2013	-2.82	-3.07,-2.57	--	--	--	--	--	--	-2.00^d^	-2.20, -1.80

^a^Joinpoint analyses with up to 3 joinpoints yielding up to 4 trends segments (Trends1-4) were based on rates per 100,0000 population and were age-adjusted to the 2000 US standard population.

^b^The APC is based on age-adjusted rates to the 2000 US standard population.

^c^The AAPC is a weighted average of the APCs that is calculated by joinpoint regression.

^d^Significantly different from 0.

Our analysis of the trends in HD mortality among adult Mississippians revealed a significant decrease from 1980 through 2013, which is consistent with a nationwide decrease from 1973 through 2010 for U.S. adults [16]. While the AAPC in HD mortality rates for Mississippians from 1980 to 2013 (-1.6%) was less than half the national AAPC for 2000 to 2010 (-3.8%) [14], the decrease is nonetheless encouraging given Mississippi’s persistent status as the state with the highest rate of HD mortality in the nation. While the declines in HD mortality among Mississippi adults are consistent with findings for U.S. adults overall [11, 14, 15, 16] the magnitude is smaller in Mississippi [14]. This difference in magnitude may be the result of the state’s prevalence of major risk factors for heart disease (e.g., high blood pressure) being perennially higher than the national level. In addition, Mississippi has a high proportion of black residents (37.0%) [3], and major HD risk factors such as high blood pressure and diabetes disproportionately affect black individuals [1]. The relatively smaller magnitude of the decline in HD mortality in Mississippi should be a red flag to health professionals in the state—a possible signal of a future increase. During the focal period, Mississippi consistently led the nation in obesity and type 2 diabetes prevalence, and the prevalence of smoking and high cholesterol were higher than the national averages [20]. In a recent study of cardiovascular disease (CVD) risk factors in the 18-county Mississippi Delta region (a region with a disproportionately high CVD burden) we found statistically significant increases in the prevalence of high cholesterol, diabetes, and obesity [21]. Decades of increases in obesity and diabetes could result in a future increase in CVD mortality [22]. In the U.S., 47% of the decline CHD deaths from 1980–2000 was attributed to evidenced-based medical therapies and 44% was attributed to changes in risk factors [4]. Factors such as a decrease in cigarette smoking, decreases in mean blood pressure and cholesterol levels, improvements in medical care, and changes in diet were found to be responsible for the decline in HD and stroke deaths from 1900 to 1999[12]. During this time period, the country implemented national efforts to reduce HD mortality; for example, the Million Hearts initiative, which began in 2012 and aims to prevent one million heart attacks by 2017 [23]. In addition, recent state initiatives have targeted high-burden, underserved, rural areas for HD prevention; one such initiative is the Mississippi Delta Health Collaborative, which serves an 18-county Mississippi Delta region (with a disproportionately high burden of HD). This initiative began in 2010 and aims to prevent HD and stroke and related chronic diseases by focusing on the “ABCS” (aspirin for those at risk, blood pressure control, cholesterol management and smoking cessation) strategy for HD and stroke prevention [21]. Our analysis demonstrated that HD mortality declined significantly for both blacks and whites between 1980 and 2013. However, the magnitude of decline was smaller for blacks. Cardiovascular health disparities between blacks and whites are well documented [24, 25]. For example, blacks are two to three times more likely to die of HD than whites [26]. Social determinants of health disparities such as education, income and income inequality, employment, racism, social networks, risk factor awareness, disease control, access to healthcare, and quality of medical care are associated with higher prevalence of CVD risk factors, morbidity, and mortality [13, 24, 26–29]. In Mississippi, the prevalence of major HD risk factors such as high blood pressure, physical inactivity, obesity, and diabetes are disproportionately higher among blacks than whites [30]. For example, in the 2013 Behavioral Risk Factor Surveillance Systems (BRFSS), blacks had a significantly higher prevalence than whites of both diabetes (14.6% vs. 12.4%) and obesity (43.3% vs. 31.8%). These significant differences in risk factor prevalence may explain the observed racial difference in the magnitude of the decline in HD rates in the state. In addition, the observed magnitude of the HD decline may be attributed to disparities in social determinants of health such as education, income, employment, access to healthcare, and geographical location within the state. For example, data from the 2013 BRFSS show that among Mississippi adults, relative to whites, blacks report higher proportions of not completing high school (24.9% vs. 15.9%), having an annual household income of less than $10,000 (19.6% vs. 6.3%), and being unemployed (11.2% vs. 5.5%) [30]. Strategies to prevent HD in Mississippi—particularly those aimed at addressing HD disparities—must include programs to address the social conditions that influence exposure and individual behavior throughout the life course [31].

Among Mississippi residents age 25 and older, HD mortality experienced significant annual declines from 1980 to 2013, but the magnitudes of these declines were lower for women than for men. Gender differences in cultural, social, and behavioral characteristics; socioeconomic status; and biological factors have been linked to the gender gap in CVD mortality rates [32, 33]. For example, in the 2013 BRFSS, men had a significantly higher prevalence of smoking than women (28.0% vs. 22.0%) while women had a significantly higher prevalence of physical inactivity than men (42.3% vs. 33.5%) [30]. From 1980 through 2013, HD mortality rates saw significant annual declines for black men, white men, black women, and white women age 25 and older. The magnitude of decline was smallest for black and white women and highest for white men. The decline among black men was slightly larger than the decline among white and black women but smaller than the decline among white men. In Mississippi in 2013, the prevalence of the primary risk factor for HD (e.g., high blood pressure) was higher among black men (42.1%) than white men (37.5%), and higher among black women (46.1%) than white women (37.0%) [30].

These findings have potential limitations. First, reliance on death certificates may introduce bias due to the misclassification of the underlying cause of death [34, 35] or the decedent’s race, [36] which could impact the conclusions drawn from epidemical studies [37]. However, death certificates are the only data source currently available to assess population trends in heart disease mortality (provided that accuracy does not vary over time) [35], and they allow the description of patterns in the whole population rather than a sample [38]. Second, changes in coding from ICD-9 to ICD-10 may affect the quality of death certificate data, however previous studies have validated the comparability of ICD-9 and ICD-10 in analyzing mortality trends [39, 5]. Finally, findings from a study of coronary heart disease (CHD) deaths in New York City hospitals showed that CHD was overreported as a cause of death on death certificates [40]. The extensive period of study and the analysis of population subgroups are key strengths of the study.

## Conclusions

In conclusion, during the past three decades, HD mortality among adult Mississippians age 25 and older has significantly declined for men, women, black men, black women, white men, and white women. However, the annual magnitude of this decline varied by race and sex. These findings should alert Mississippi public health professionals and policymakers to continually and increasingly support preventive efforts aimed at the major HD risk factors. Such efforts could sustain the decline, particularly among populations who have experienced smaller decreases in HD mortality. Ongoing collaborative efforts between the Centers for Disease Control and Prevention and the Mississippi State Department of Health (through the Mississippi Delta Health Collaborative initiative, MDHC) to implement programs aimed at preventing and reducing the risks of HD and stroke in the Mississippi Delta region (www.healthyms.com/MDHC)—a region with a high HD burden—are a step in the right direction.

## Supporting Information

S1 Fig Overall trends in heart disease age-adjusted mortality rates among Mississippi adults, 25 years and older, 1980–2013. (JPG)

S2 Fig Trends in heart disease age-adjusted mortality rates among Mississippi Black adults, 25 years and older, 1980–2013. (JPG)

S3 Fig Trends in heart disease age-adjusted mortality rates among Mississippi White adults, 25 years and older, 1980–2013. (JPG)

S4 Fig Trends in heart disease age-adjusted mortality rates among Mississippi Black Female adults, 25 years and older, 1980–2013. (JPG)

S5 Fig Trends in heart disease age-adjusted mortality rates among Mississippi Black Male adults, 25 years and older, 1980–2013. (JPG)

S6 Fig Trends in heart disease age-adjusted mortality rates among Mississippi White Female adults, 25 years and older, 1980–2013. (JPG)

S7 Fig Trends in heart disease age-adjusted mortality rates among Mississippi White Male adults, 25 years and older, 1980–2013. (JPG)

S8 Fig Trends in heart disease age-adjusted mortality rates among Mississippi Female adults, 25 years and older, 1980–2013. (JPG)

S9 Fig Trends in heart disease age-adjusted mortality rates among Mississippi Male adults, 25 years and older, 1980–2013. (JPG)

## References

[1] Mozaffarian D, Benjamin EJ, Go AS, Arnett DK, Blaha MJ, Cushman M, et al Heart Disease and Stroke Statistics-2016 Update: A Report From the American Heart Association. Circulation. 2015;132:000–000. 10.1161/CIR.0000000000000350 26673558

[2] Centers for Disease Control and Prevention, National Center for Health Statistics. Underlying Cause of Death 1999–2014 on CDC WONDER Online Database, released 2015. Data are from the Multiple Cause of Death Files, 1999–2014, as compiled from data provided by the 57 vital statistics jurisdictions through the Vital Statistics Cooperative Program. Available: http://wonder.cdc.gov/ucd-icd10.html. Accessed at April 24, 2015.

[3] Mississippi Vital Statistics (The Mississippi STatistically Automated Health Resource System (MSTAHRS), 2013. Available: http://mstahrs.msdh.ms.gov/. Accessed April 23, 2015.

[4] Ford ES, Ajani UA, Croft JB, Critchley JA, Labarthe DR, Kottke TE, et al Explaining the decrease in U.S. deaths from coronary disease, 1980–2000. N Engl J Med. 2007;356(23):2388–98. 17554120 10.1056/NEJMsa053935

[5] Rosamond WD, Chambless LE, Heiss G, Mosley TH, Coresh J, Whitsel E, et al Twenty-two-year trends in incidence of myocardial infarction, coronary heart disease mortality, and case fatality in 4 US communities, 1987–2008. Circulation. 2012;125(15):1848–57. 10.1161/CIRCULATIONAHA.111.047480 22420957 PMC3341729

[6] O'flaherty M, Allender S, Taylor R, Stevenson C, Peeters A, Capewell S. The decline in coronary heart disease mortality is slowing in young adults (Australia 1976–2006): a time trend analysis. Int J Cardiol. 2012;158(2):193–8. 10.1016/j.ijcard.2011.01.016 21288580

[7] O'flaherty M, Ford E, Allender S, Scarborough P, Capewell S. Coronary heart disease trends in England and Wales from 1984 to 2004: concealed levelling of mortality rates among young adults. Heart. 2008;94(2):178–81. 17641070 10.1136/hrt.2007.118323

[8] Berg J, Björck L, Lappas G, O'flaherty M, Capewell S, Rosengren A. Continuing decrease in coronary heart disease mortality in Sweden. BMC Cardiovasc Disord. 2014;14:9 10.1186/1471-2261-14-9 24447603 PMC3930358

[9] Unal B, Sözmen K, Arık H, Gerçeklioğlu G, Altun DU, Şimşek H, et al Explaining the decline in coronary heart disease mortality in Turkey between 1995 and 2008. BMC Public Health. 2013;13:1135 10.1186/1471-2458-13-1135 24308515 PMC4234124

[10] Nichols M, Townsend N, Scarborough P, Rayner M. Trends in age-specific coronary heart disease mortality in the European Union over three decades: 1980–2009. Eur Heart J. 2013;34(39):3017–27. 10.1093/eurheartj/eht159 23801825 PMC3796269

[11] Ford ES, Capewell S. Coronary heart disease mortality among young adults in the U.S. from 1980 through 2002: concealed leveling of mortality rates. J Am Coll Cardiol. 2007;50(22):2128–32. 18036449 10.1016/j.jacc.2007.05.056

[12] Decline in deaths from heart disease and stroke—United States, 1900–1999. MMWR Morb Mortal Wkly Rep. 1999;48(30):649–56. 10488780

[13] Cooper R, Cutler J, Desvigne-nickens P, Fortmann SP, Friedman L, Havlik R, et al Trends and disparities in coronary heart disease, stroke, and other cardiovascular diseases in the United States: findings of the national conference on cardiovascular disease prevention. Circulation. 2000;102(25):3137–47. 11120707 10.1161/01.cir.102.25.3137

[14] Hong Y; Ritchey M; Loustalot F; Bowman BA. Heart Disease Mortality Trends During the 21st Century, United States, 2000–2010. [Abstract] Circulation. 2013; 128: A13132.

[15] Ritchey MD, Loustalot F, Bowman BA, Hong Y. Trends in mortality rates by subtypes of heart disease in the United States, 2000–2010. JAMA. 2014;312(19):2037–9. 10.1001/jama.2014.11344 25399281 PMC6956256

[16] Vaughan AS, Quick H, Pathak EB, Kramer MR, Casper M. Disparities in Temporal and Geographic Patterns of Declining Heart Disease Mortality by Race and Sex in the United States, 1973–2010. J Am Heart Assoc. 2015;4(12). 10.1161/JAHA.115.002567 PMC4845281 26672077

[17] Edwards BK, Noone AM, Mariotto AB, Henley SJ, Jemal A, Cho H, et al Annual Report to the Nation on the status of cancer, 1975–2010, featuring prevalence of comorbidity and impact on survival among persons with lung, colorectal, breast, or prostate cancer. Cancer. 2014;120(9):1290–314. 10.1002/cncr.28509 24343171 PMC3999205

[18] Cayuela A, Rodríguez-Domínguez S, López-Campos JL, Otero Candelera R, Rodríguez Matutes C. Joinpoint regression analysis of lung cancer mortality, Andalusia 1975–2000. Ann Oncol 2004;15(5):793–6. 15111349 10.1093/annonc/mdh170

[19] Qiu D, Katanoda K, Marugame T, Sobue T. A joinpoint regression analysis of long-term trends in cancer mortality inJapan (1958–2004). Int J Cancer 2009;124(2):443–8. 10.1002/ijc.23911 18844218

[20] Mississippi State Department of Health, 2009–2013 Behavioral Risk Factor Surveillance System, Available: http://www.cdc.gov/brfss/brfssprevalence/index.html. Accessed August 18, 2015.

[21] Mendy VL, Vargas R. Trends in major risk factors for cardiovascular disease among adults in the Mississippi Delta region, Mississippi Behavioral Risk Factor Surveillance System, 2001–2010. Prev Chronic Dis. 2015;12:E21 10.5888/pcd12.140481 25695259 PMC4335616

[22] Capewell S, Buchan I. Why have sustained increases in obesity and type 2 diabetes not offset declines in cardiovascular mortality over recent decades in Western countries?. Nutr Metab Cardiovasc Dis. 2012;22(4):307–11. 10.1016/j.numecd.2012.01.005 22405577

[23] Frieden TR, Berwick DM. The "Million Hearts" initiative—preventing heart attacks and strokes. N Engl J Med. 2011;365(13):e27 10.1056/NEJMp1110421 21913835

[24] Mensah GA, Mokdad AH, Ford ES, Greenlund KJ, Croft JB. State of disparities in cardiovascular health in the United States. Circulation. 2005;111(10):1233–41. 15769763 10.1161/01.CIR.0000158136.76824.04

[25] Davis AM, Vinci LM, Okwuosa TM, Chase AR, Huang ES. Cardiovascular health disparities: a systematic review of health care interventions. Med Care Res Rev. 2007;64(5 Suppl):29S–100S. 17881625 10.1177/1077558707305416 PMC2367222

[26] Havranek EP, Mujahid MS, Barr DA, Blair IV, Cohen MS, Cruz-Flores S, Davey-Smith G, Dennison-Himmelfarb CR, Lauer MS, Lockwood DW, Rosal M, Yancy CW. Social Determinants of Risk and Outcomes for Cardiovascular Disease: A Scientific Statement From the American Heart Association. Circulation. 2015;132(9):873–98. 10.1161/CIR.0000000000000228 26240271

[27] Christian AH, Rosamond W, White AR, Mosca L. Nine-year trends and racial and ethnic disparities in women's awareness of heart disease and stroke: an American Heart Association national study. J Womens Health (Larchmt). 2007;16(1):68–81. 17274739 10.1089/jwh.2006.M072

[28] Mcwilliams JM, Meara E, Zaslavsky AM, Ayanian JZ. Differences in control of cardiovascular disease and diabetes by race, ethnicity, and education: U.S. trends from 1999 to 2006 and effects of medicare coverage. Ann Intern Med. 2009;150(8):505–15. 19380852 10.7326/0003-4819-150-8-200904210-00005

[29] Rooks RN, Simonsick EM, Klesges LM, Newman AB, Ayonayon HN, Harris TB. Racial disparities in health care access and cardiovascular disease indicators in Black and White older adults in the Health ABC Study. J Aging Health. 2008;20(6):599–614. 10.1177/0898264308321023 18625758 PMC2733332

[30] Mississippi State Department of Health. 2013 Behavioral Risk Factor Surveillance System. Available: http://www.msdh.state.ms.us/brfss/. Accessed August 18, 2015.

[31] Marmot M. The health gap: the challenge of an unequal world. Lancet. 2015;386(10011):2442–4. 10.1016/S0140-6736(15)00150-6 26364261

[32] Pilote L, Dasgupta K, Guru V, Humphries KH, McGrath J, Norris C, et al A comprehensive view of sex-specific issues related to cardiovascular disease. CMAJ. 2007;176(6):S1–44. 17353516 10.1503/cmaj.051455 PMC1817670

[33] Rogers RG, Everett BG, Onge JM, Krueger PM. Social, behavioral, and biological factors, and sex differences in mortality. Demography. 2010;47(3):555–78. 20879677 10.1353/dem.0.0119 PMC3000060

[34] Wexelman BA, Eden E, Rose KM. Survey of New York City Resident Physicians on Cause-of-Death Reporting, 2010. Prev Chronic Dis 2013;10:120288. 10.5888/pcd10.120288 PMC3664206 23660118

[35] Gerber Y, Jacobsen SJ, Frye RL, Weston SA, Killian JM, Roger VL. Secular trends in deaths from cardiovascular diseases: a 25-year community study. Circulation. 2006;113(19):2285–92. 16682616 10.1161/CIRCULATIONAHA.105.590463

[36] Horner RD, Day GM, Lanier AP, Provost EM, Hamel RD, Trimble BA. Stroke mortality among Alaska Native people. Am J Public Health. 2009;99(11):1996–2000. 10.2105/AJPH.2008.148221 19762671 PMC2759800

[37] Harriss LR, Ajani AE, Hunt D, Shaw J, Chambers B, Dewey H, et al Accuracy of national mortality codes in identifying adjudicated cardiovascular deaths. Aust N Z J Public Health. 2011;35(5):466–76. 10.1111/j.1753-6405.2011.00739.x 21973254

[38] Cohen J, Bilsen J, Miccinesi G, Lofmark R, Addington-Hall J, Kaasa S, et al Using death certificate data to study place of death in 9 European countries: opportunities and weaknesses. BMC Public Health. 2007;7:283 17922894 10.1186/1471-2458-7-283 PMC2099436

[39] Anderson RN, Miniño AM, Hoyert DL, Rosenberg HM. Comparability of cause of death between ICD-9 and ICD-10: preliminary estimates. Natl Vital Stat Rep. 2001;49(2):1–32. 11381674

[40] Agarwal R, Norton JM, Konty K, Zimmerman R, Glover M, Lekiachvili A, et al Overreporting of deaths from coronary heart disease in New York City hospitals, 2003. Prev Chronic Dis. 2010;7(3):A47 20394686 PMC2879979

