# Supplementary figures and images for: Trends in Heart Disease Mortality among Mississippi Adults over Three Decades, 1980-2013
Source: PLoS One. 2016 Aug 12;11(8):e0161194. doi: 10.1371/journal.pone.0161194 (PMC4982678; doi:10.1371/journal.pone.0161194)

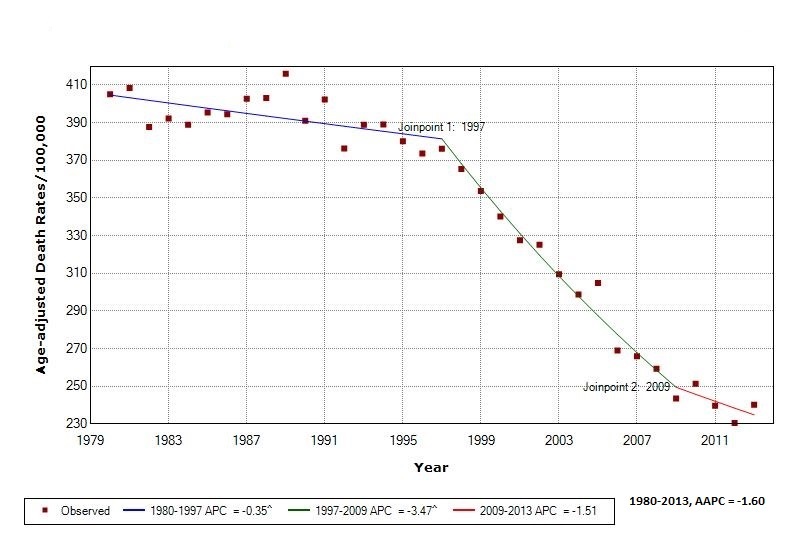

Supplement: S1 Fig — (JPG) [file pone.0161194.s001.jpg]

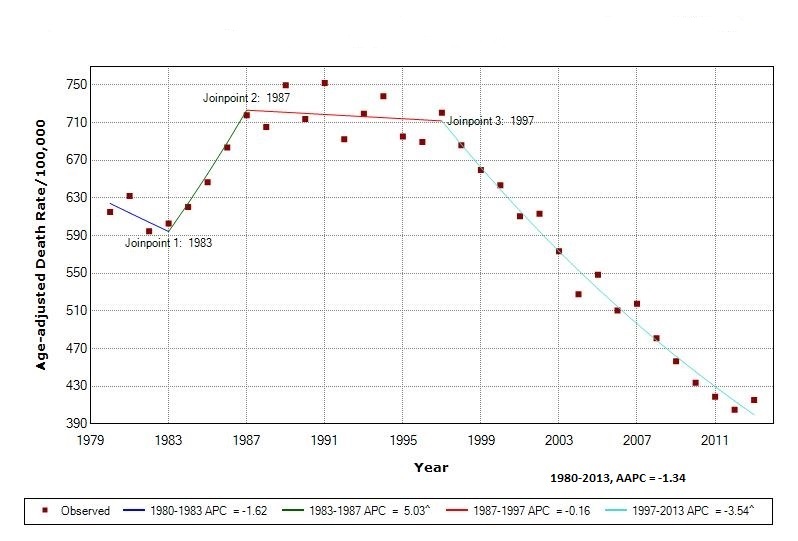

Supplement: S2 Fig — (JPG) [file pone.0161194.s002.jpg]

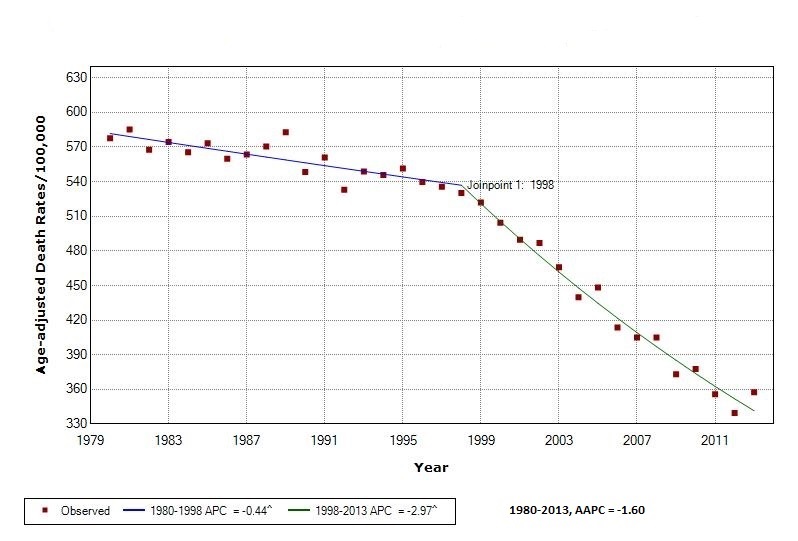

Supplement: S3 Fig — (JPG) [file pone.0161194.s003.jpg]

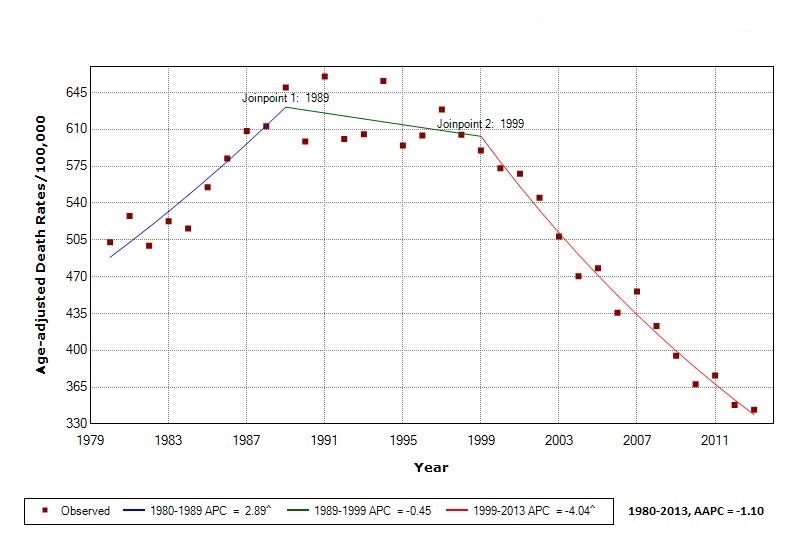

Supplement: S4 Fig — (JPG) [file pone.0161194.s004.jpg]

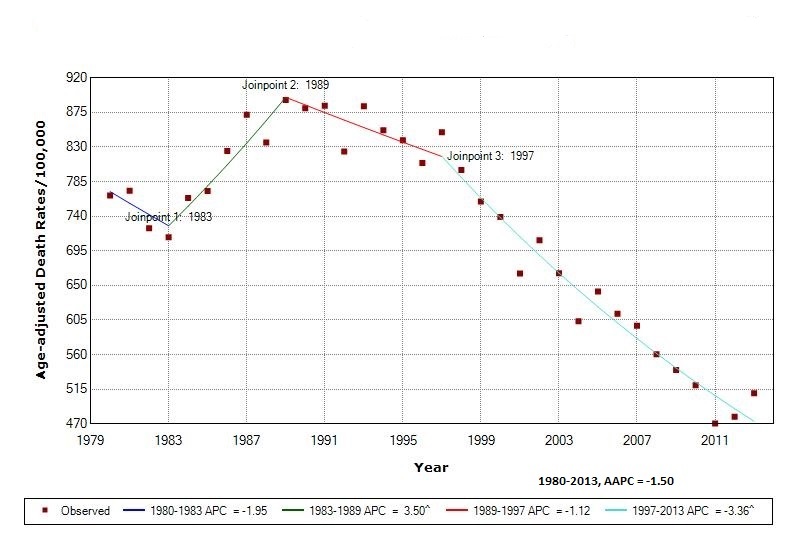

Supplement: S5 Fig — (JPG) [file pone.0161194.s005.jpg]

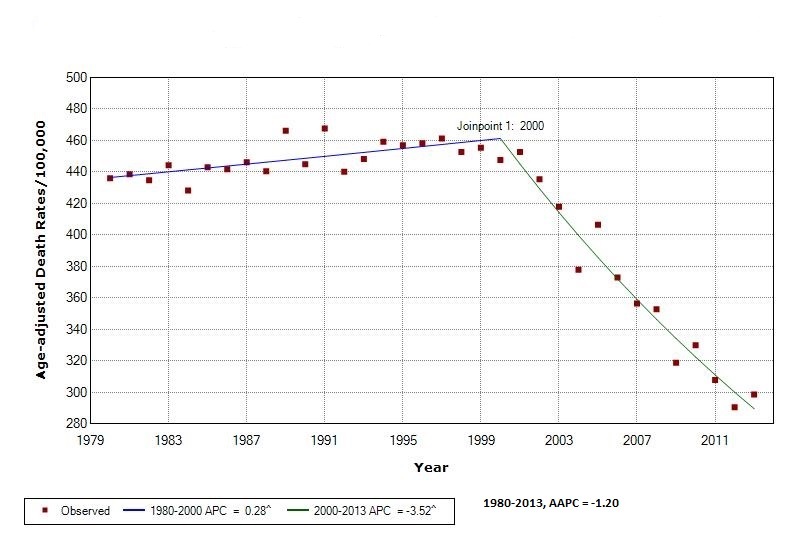

Supplement: S6 Fig — (JPG) [file pone.0161194.s006.jpg]

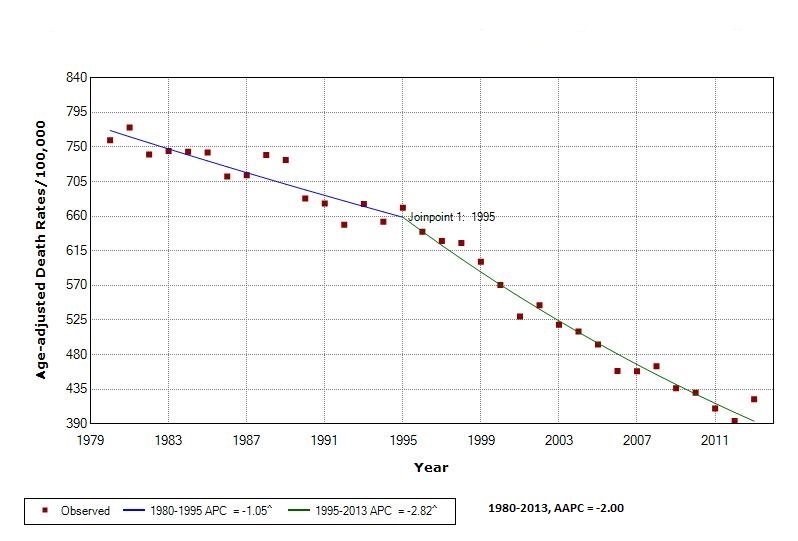

Supplement: S7 Fig — (JPG) [file pone.0161194.s007.jpg]

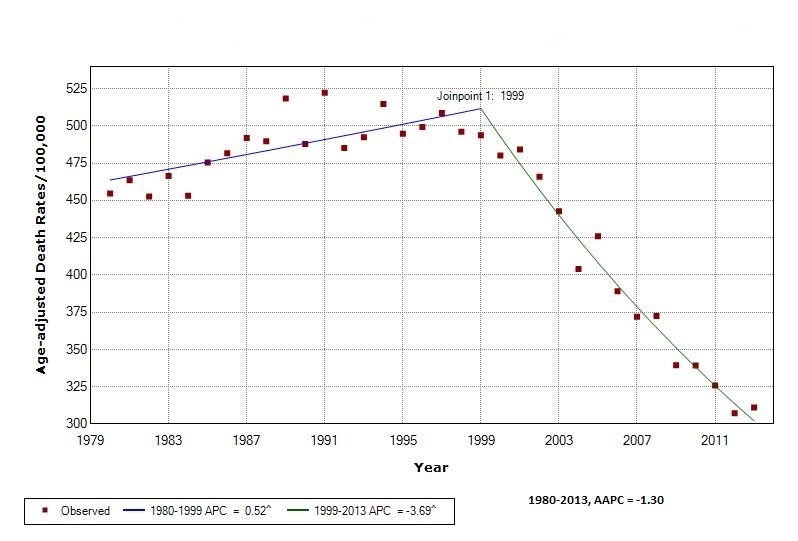

Supplement: S8 Fig — (JPG) [file pone.0161194.s008.jpg]

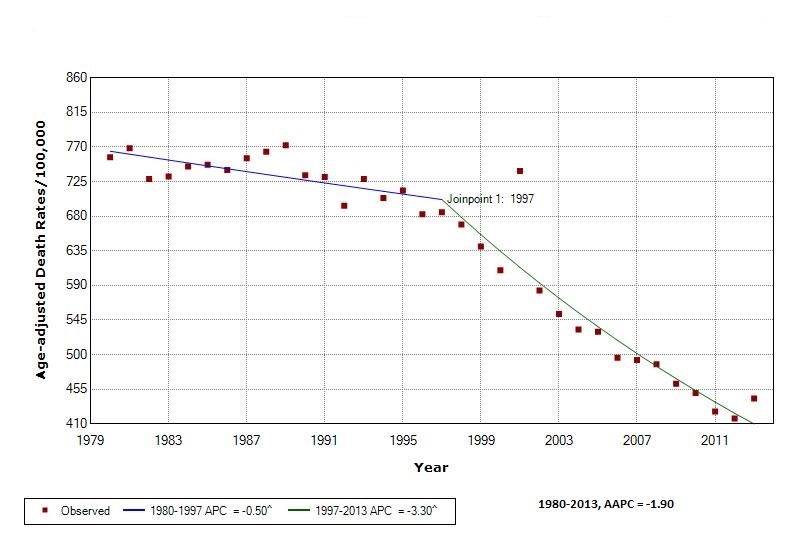

Supplement: S9 Fig — (JPG) [file pone.0161194.s009.jpg]
